# Causal relationship between rheumatoid arthritis and ankylosing spondylitis: Two-sample Mendelian randomization

**DOI:** 10.1097/MD.0000000000039132

**Published:** 2024-07-26

**Authors:** Guang-hua Deng

**Affiliations:** a Ya’an Hospital of Traditional Chinese Medicine, Orthopaedic Clinic, Sichuan, China.

**Keywords:** ankylosing spondylitis, Mendelian randomization, rheumatoid arthritis

## Abstract

To investigate the causal relationship between rheumatoid arthritis (RA) and ankylosing spondylitis using Mendelian randomization (MR). Genetic loci independently associated with RA and ankylosing spondylitis in people of European origin were selected as instrumental variables using pooled data from large-scale genome-wide association studies. Three MR analyses, MR-Egger, weighted median, and inverse variance weighting, were used to investigate the causal relationship between RA and ankylosing spondylitis. Heterogeneity and multiplicity tests were used, and a sensitivity test using the “leave-one-out” method was used to explore the robustness of the results. The inverse variance weighting results showed an OR (95 % CI) of 1.25 (1.11–1.41), *P* < .001, indicating a causal relationship between RA and ankylosing spondylitis. And no heterogeneity and pleiotropy were found by the test and sensitivity analysis also showed robust results. The present study was conducted to analyze and explore the genetic data using two-sample MR analysis and the results showed that there is a causal relationship between RA and the occurrence of ankylosing spondylitis.

## 1. Introduction

Ankylosing spondylitis is a chronic inflammatory joint disease, which is an autoimmune disease with a genetic predisposition.^[1]^ Ankylosing spondylitis is most commonly characterized by lumbosacral pain and stiffness.^[2]^ In advanced stages of the disease, the spine may become curved and stiff.^[3]^ This curvature may lead to changes in body posture and reduction in height. The deformity and dysfunction of the spine may affect the patient’s normal life and work.^[4]^ In recent years, many studies have found that rheumatoid arthritis (RA) is associated with the development of several diseases, such as neck pain^[5]^ and foot pain.^[6]^ And some studies have found that patients with RA are more likely to suffer from ankylosing spondylitis.^[7]^ However, studies on RA and ankylosing spondylitis are relatively lacking, and the causal relationship is uncertain. The causal relationship between RA and frozen shoulder still needs further investigation.

The association between RA and ankylosing spondylitis may be influenced to some extent by confounding factors and reverse causality inherent in traditional observational studies.^[8]^ In contrast, Mendelian randomization (MR), a genetic epidemiological method, is a useful tool for assessing the causal role of RA and ankylosing spondylitis.^[9]^ By using genetic variants such as single nucleotide polymorphisms (SNPs) as instrumental variants that can modify disease risk factors or exposures, MR studies can enhance causal inference of exposure–outcome associations.^[10]^ According to Mendel laws of inheritance, genetic variants are not susceptible to confounding factors because they are randomly assigned during gamete formation.^[11]^ In addition, confounders and reverse causation can be minimized as genotypes cannot change as the disease progresses.^[12]^

To this end, we conducted a two-sample MR study to examine the causal relationship between RA and ankylosing spondylitis. We aimed to provide significant evidence for the causal role of RA in causing ankylosing spondylitis.

## 2. Data and methods

### 2.1. Data sources

Genome-wide association studies (GWAS) data for RA and ankylosing spondylitis were obtained via the IEU Open GWAS project (mr cieu.ac.uk) website. The website was accessed on September 10, 2023.The population source for all final data was European, male, and female. Including RA (ebi-a-GCST90013534) containing 13,108,512 SNPs with a sample size of 58,284 and ankylosing spondylitis (finn-b-M13_ANKYLOSPON) containing 16,380,022 SNPs with 1462 in the test group and 164,682 in the control group. This study was a reanalysis of previously collected and published data and therefore did not require additional ethical approval.

### 2.2. Conditioning of SNP as an instrumental variable

Firstly, the instrumental variables were highly correlated with exposure, with F > 10 as a strong correlation criterion.^[13]^ Second, the instrumental variable is not directly related to the outcome, but only affects the outcome through exposure, that is, there is no genetic pleiotropy. In this study, the nonexistence of genetic pleiotropy was indicated by a nonzero intercept term (*P* < .05) in the MR-Egger regression model.^[14]^ Finally, instrumental variables were not associated with untested confounding.^[15]^ The human genotype–phenotype association database Phenoscanner V2 was searched for phenotypes associated with the instrumental variables at the genome-wide significance level to determine whether these SNPs were associated with potential risk factors.^[16]^

### 2.3. SNP screening

Significant SNPs were screened from the GWAS pooled data of RA (*P* < 5 × 10^‐8^ was used as the screening condition)^[17]^; the chain disequilibrium coefficient r2 was set to be 0.001, and the width of chain disequilibrium region to be 10,000 kb to ensure that individual SNPs were independent of each other.^[18]^ The RA-associated SNPs screened above were extracted from the GWAS pooled data of ankylosing spondylitis, while SNPs directly associated with outcome indicators were excluded (*P* < 5 × 10^‐8^). The F-value of each SNP was calculated, and SNPs with weak instrumental variables (F-value <10) were excluded.^[19]^ And the human genotype–phenotype association database was queried to screen for potentially relevant risk factor SNPs and exclude them.^[20]^

### 2.4. Causality validation methods

The causal relationship between exposure (RA) and outcome (ankylosing spondylitis) was mainly verified using inverse variance weighted (IVW) as, supplemented by 3 MR analyses, namely MR-Egger and weighted median, with SNPs as instrumental variables.

### 2.5. Sensitivity analysis

Sensitivity analyses were conducted using several methods. First, the Cochran *Q* test was used to assess the heterogeneity between individual SNP estimates, and a statistically significant Cochran *Q* test demonstrated significant heterogeneity in the analyses. Second, MR pleiotropy residual sum and outlier (MR-PRESSO) was used to validate the results in the IVW model, to correct for the effect of outliers, and to exclude outliers if they existed and rerun the analysis. Third, the horizontal multiplicity of SNPs was tested using the MR-Egger intercept test, and if the intercept term in the MR-Egger intercept test analysis was statistically significant, it indicated that the MR analyses had significant horizontal multiplicity. Fourth, “leave-one-out” sensitivity analyses were performed by removing a single SNP at a time to assess whether the variant drove the association between the exposure and outcome variables. Fifth, funnel plots and forest plots were constructed to visualize the results of the sensitivity analyses. *P* < .05 suggests that there is a potential causal relationship in the MR analyses, which is statistically significant. All statistical analyses were performed using the “TwoSampleMR” package in R software version 4.3.0.

## 3. Results

### 3.1. Instrumental variables

In this study, 90 SNPs that were closely associated with RA (*P* < 5 × 10^‐8^) without chain imbalance (r2 < 0.001, kb = 10,000) were screened out. 86 SNPs were left by taking the intersection with SNPs in the pooled data from GWAS for ankylosing spondylitis, and 86 SNPs were left by excluding SNPs that were directly associated with the outcome metrics. In our study, the SNPs for each F-value was >10, indicating no weak instrumental variables (Table 1). We searched the human genotype–phenotype association database and found no potentially relevant risk factor SNPs.

**Table 1:** Information on the final screening of rheumatoid arthritis SNPs from GWAS data (n = 86).

ID	SNP	Effect_Allele	Other_Allele	β	SE	P	F
1	rs10435844	T	G	‐0.0784	0.0121	9.73E-11	41
2	rs10911902	T	C	‐0.0847	0.0152	2.36E-08	31
3	rs11123811	C	T	‐0.0995	0.0114	2.01E-18	76
4	rs112733823	T	C	0.191	0.0188	3.82E-24	103
5	rs114508013	A	G	0.488	0.0441	1.82E-28	122
6	rs115521560	C	A	0.7878	0.0364	1.29E-103	468
7	rs11574914	A	G	0.1153	0.0149	9.92E-15	59
8	rs117026326	T	C	0.381	0.0424	2.45E-19	80
9	rs11754264	C	T	‐0.1359	0.0193	1.88E-12	49
10	rs11889341	T	C	0.1466	0.0129	4.32E-30	129
11	rs12126142	A	G	‐0.0751	0.0116	1.01E-10	41
12	rs1234313	G	A	0.0797	0.0133	1.90E-09	35
13	rs12466919	T	C	0.1025	0.0152	1.59E-11	45
14	rs12530098	T	C	0.1382	0.0204	1.35E-11	45
15	rs12918327	T	C	0.0867	0.0157	3.04E-08	30
16	rs13103285	T	C	0.0989	0.0131	4.29E-14	56
17	rs1355208	G	A	0.0818	0.0119	6.77E-12	47
18	rs139395255	G	A	0.3833	0.0235	8.68E-60	266
19	rs146305655	A	G	‐0.4379	0.0452	3.29E-22	93
20	rs1538981	T	C	0.0671	0.0114	4.42E-09	34
21	rs1571878	T	C	‐0.1539	0.0116	4.13E-40	176
22	rs1595260	T	A	0.0845	0.0126	2.29E-11	44
23	rs1611236	A	G	‐0.1165	0.0131	4.54E-19	79
24	rs1858037	A	T	‐0.1012	0.0131	1.14E-14	59
25	rs1883832	C	T	0.1052	0.0127	1.13E-16	68
26	rs1893592	C	A	‐0.0976	0.0132	1.48E-13	54
27	rs1950897	T	C	0.1069	0.0144	1.02E-13	55
28	rs2069235	A	G	0.1296	0.014	1.69E-20	85
29	rs2073609	C	T	0.1029	0.0182	1.47E-08	31
30	rs2076616	G	A	‐0.0885	0.0135	6.20E-11	42
31	rs212389	A	G	0.1058	0.0147	6.66E-13	51
32	rs2233424	T	C	0.1964	0.0187	6.49E-26	110
33	rs2258734	A	G	‐0.0921	0.0123	6.04E-14	56
34	rs2275806	A	G	‐0.0725	0.0122	2.51E-09	35
35	rs2301888	A	G	‐0.1282	0.0121	3.75E-26	112
36	rs244685	G	T	‐0.089	0.0144	6.04E-10	38
37	rs28411352	T	C	0.0914	0.0136	1.66E-11	45
38	rs2841275	C	A	0.1617	0.0179	1.71E-19	81
39	rs28421442	A	T	‐0.1234	0.0214	7.86E-09	33
40	rs2847297	G	A	0.0903	0.0119	2.65E-14	57
41	rs2918392	C	T	0.0668	0.0122	4.62E-08	29
42	rs3025669	G	C	‐0.2534	0.0222	2.91E-30	130
43	rs3087243	A	G	‐0.1261	0.0124	3.32E-24	103
44	rs3134883	A	G	0.0991	0.0125	1.98E-15	62
45	rs34046593	A	G	0.1422	0.017	7.17E-17	69
46	rs34502849	A	G	‐0.0851	0.014	1.07E-09	36
47	rs34536443	C	G	‐0.3801	0.0474	1.08E-15	64
48	rs3757387	C	T	0.1236	0.0137	1.87E-19	81
49	rs3761959	T	C	0.0744	0.0115	9.64E-11	41
50	rs3806624	G	A	0.0863	0.0131	3.94E-11	43
51	rs403214	G	A	‐0.0914	0.0146	3.96E-10	39
52	rs42034	G	A	0.0871	0.0153	1.28E-08	32
53	rs4409785	C	T	0.0982	0.017	7.85E-09	33
54	rs4602367	G	A	0.075	0.0117	1.76E-10	41
55	rs4622308	T	C	0.0878	0.0125	2.21E-12	49
56	rs4717901	C	A	0.249	0.0349	9.52E-13	50
57	rs4795400	T	C	0.0743	0.012	5.86E-10	38
58	rs4963581	A	G	0.0856	0.0156	3.75E-08	30
59	rs5020946	T	G	0.6519	0.0169	1.00E-200	1487
60	rs502919	C	T	0.0829	0.0134	6.17E-10	38
61	rs5754104	A	G	0.0891	0.0139	1.36E-10	41
62	rs5912815	G	T	‐0.0787	0.0134	4.79E-09	34
63	rs6011186	T	C	‐0.1074	0.0171	3.19E-10	39
64	rs61828284	T	C	‐0.2018	0.0348	6.33E-09	33
65	rs62422878	T	C	0.1037	0.0176	3.57E-09	34
66	rs6421571	C	T	0.134	0.0178	5.57E-14	56
67	rs6479800	C	G	0.1202	0.0181	3.01E-11	44
68	rs660442	A	G	‐0.1067	0.0175	1.11E-09	37
69	rs6679677	A	C	0.591	0.023	1.41E-145	660
70	rs7097397	A	G	‐0.0847	0.012	1.42E-12	49
71	rs71508903	T	C	0.1487	0.0143	3.13E-25	108
72	rs71565312	A	G	0.699	0.0402	1.11E-67	302
73	rs7170107	T	C	0.1366	0.0158	6.11E-18	74
74	rs7206670	T	G	0.0701	0.0119	4.14E-09	34
75	rs740122	A	G	‐0.0782	0.0134	5.37E-09	34
76	rs76153210	T	C	0.1597	0.0205	6.83E-15	60
77	rs7731626	A	G	‐0.1956	0.0184	1.94E-26	113
78	rs7749323	A	G	0.2835	0.0253	3.47E-29	125
79	rs8032939	C	T	0.1244	0.0123	4.47E-24	102
80	rs8126756	C	T	‐0.0823	0.0137	1.81E-09	36
81	rs9271365	G	T	0.4888	0.0128	1.00E-200	1458
82	rs9405192	A	G	‐0.089	0.0137	9.26E-11	42
83	rs9532434	C	T	0.114	0.0126	1.94E-19	81
84	rs9693589	A	G	0.1127	0.0128	1.50E-18	77
85	rs9927316	G	C	0.0906	0.0136	2.30E-11	44
86	rs9943599	T	C	0.083	0.0131	2.70E-10	40

### 3.2. Causal relationship between RA and ankylosing spondylitis

**Figure 1.**
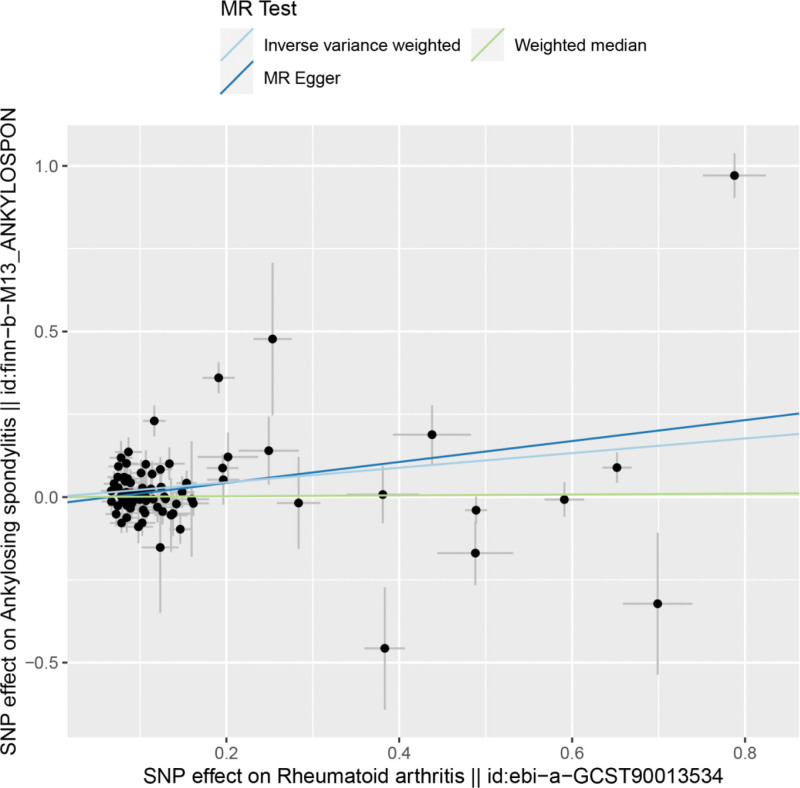
Scatter plot of rheumatoid arthritis and ankylosing spondylitis.

**Figure 2.**
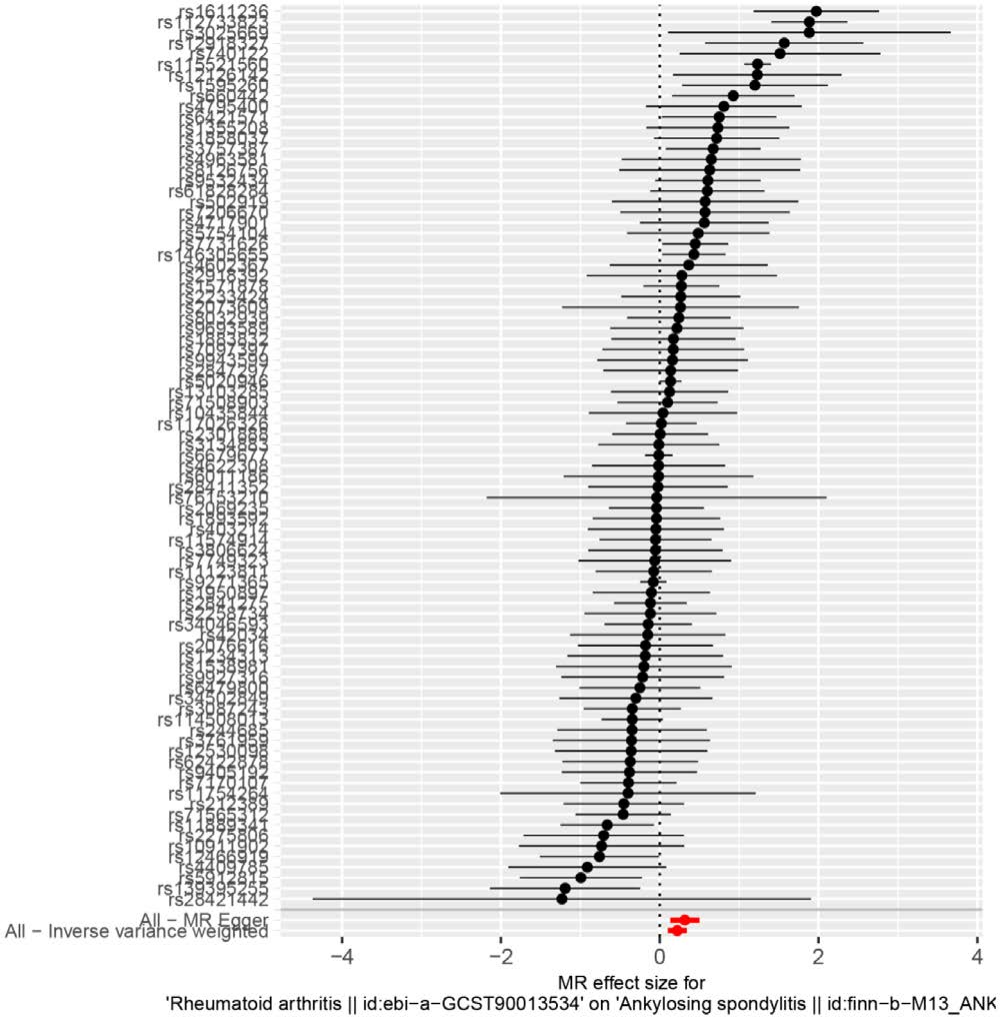
Forest plot of rheumatoid arthritis and ankylosing spondylitis.

By MR analysis, the results of both IVW and MR-Egger showed a causal relationship between RA and ankylosing spondylitis IVW: OR = 1.25, 95% CI = 1.11–1.41, *P* < .001; weighted median: OR = 1.01, 95% CI = 0.92–1.12, *P* = .800; MR-Egger: OR = 1.37, 95% CI = 1.14–1.65, *P* = .001 (see Table 2 for details). Scatter plot (Fig. 1) and forest plot (Fig. 2) also showed a causal relationship between RA and ankylosing spondylitis.

**Table 2:** MR regression results of the 3 methods.

Method	β	SE	OR (95% CI)	*P*
IVW	0.221	0.061	1.25 (1.11–1.41)	<.001
WME	0.013	0.052	1.01 (0.92–1.12)	.800
MR-Egger	0.316	0.094	1.37 (1.14–165)	.001

### 3.3. Sensitivity analysis

**Figure 3.**
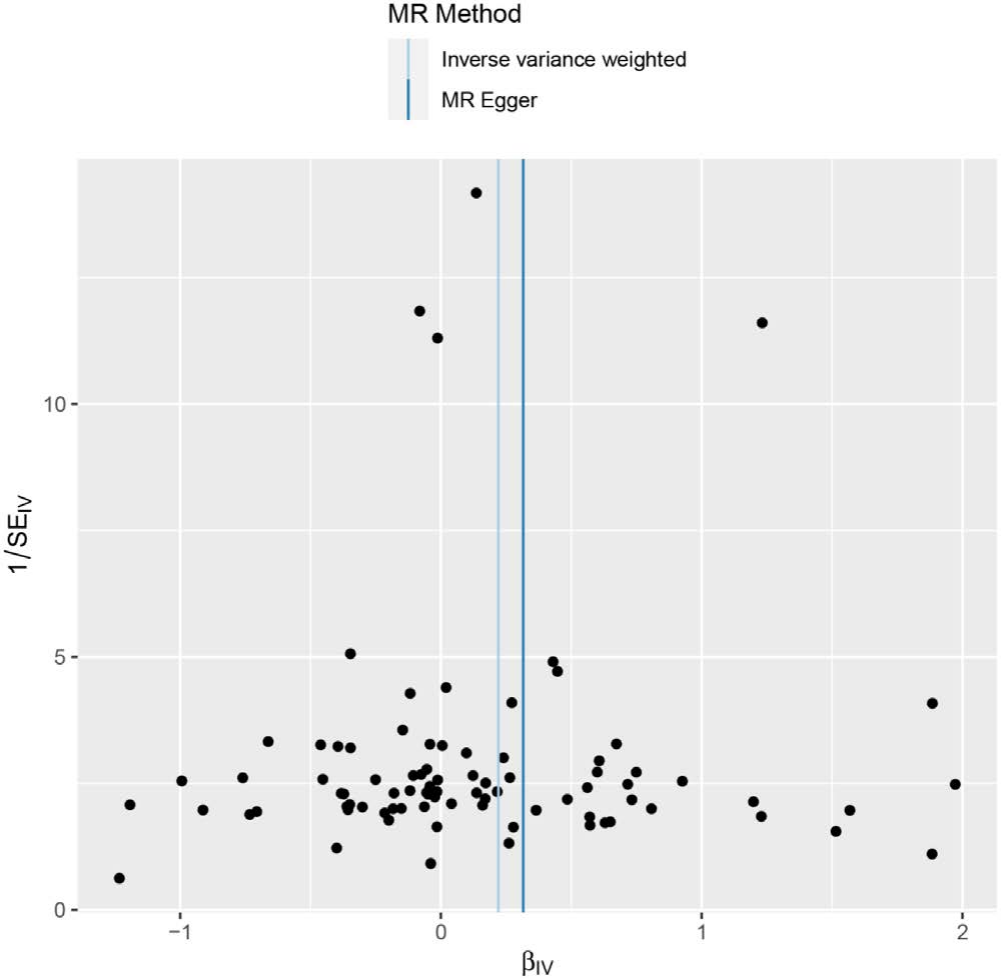
Funnel plot of rheumatoid arthritis and ankylosing spondylitis.

**Figure 4.**
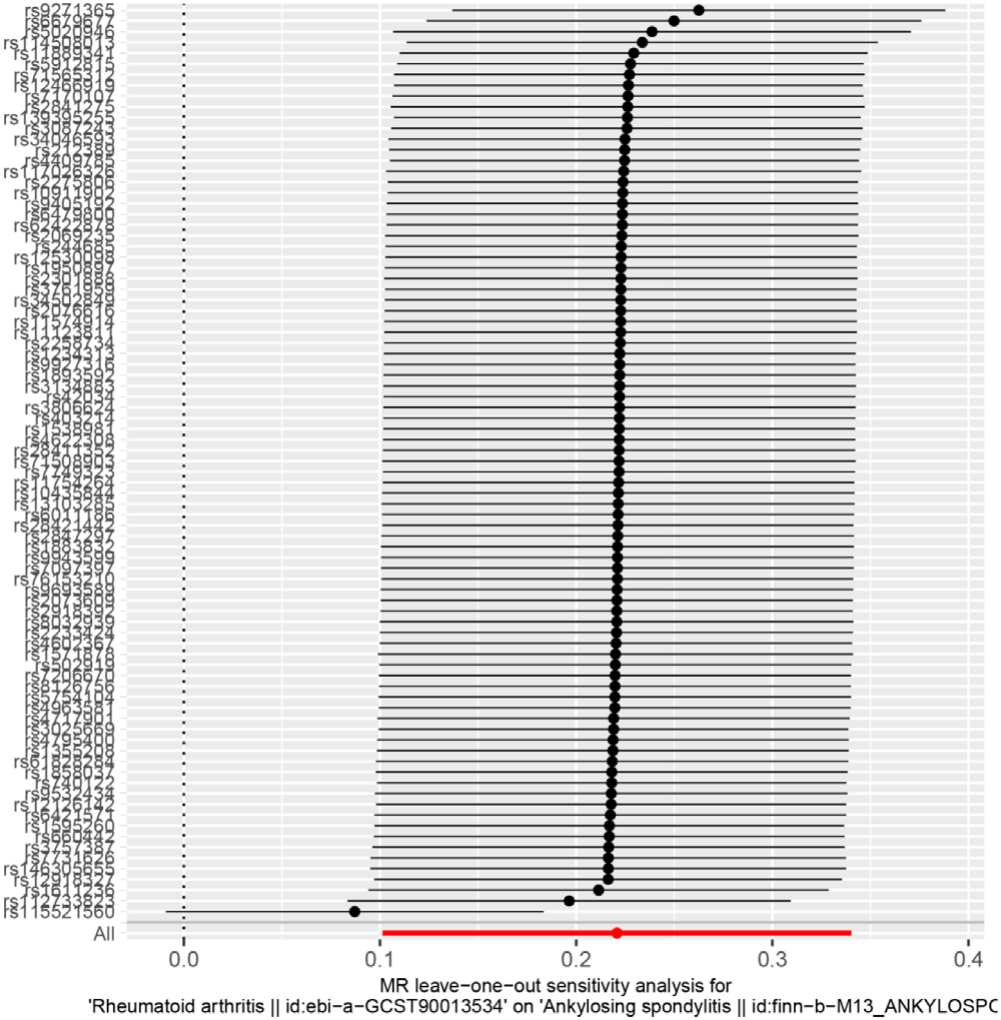
Analysis of rheumatoid arthritis and ankylosing spondylitis by the leave-one-out method.

Heterogeneity was tested using the IVW method (Cochran *Q* test, *P* = .063) and the results suggested that there was no heterogeneity. A funnel plot was drawn to show the results of heterogeneity, as shown in Figure 3. MR-PRESSO was used to screen for SNPs that could lead to heterogeneity, and the results did not reveal any SNPs that would lead to heterogeneity in the results. The result of Global test by MR-PRESSO suggested that there was no pleiotropy (*P* = .185). The “leave-one-out” method uses the IVW method by default, and as can be seen in Figure 4, no single SNP will have a large impact on the overall results after eliminating any SNP, indicating that the results are robust.

## 4. Discussion

It is known that RA may be an observational risk factor for ankylosing spondylitis, but the causality of this association is unclear. Our MR study aimed to reveal the causal relationship between RA and ankylosing spondylitis. The results showed a causal association between RA and the occurrence of ankylosing spondylitis, as demonstrated by the two-sample MR results, with an OR (95 % CI) of 1.25 (1.11–1.41), *P* < .001, suggesting that people with RA are at an increased risk of ankylosing spondylitis compared to the general population.

The exact cause of ankylosing spondylitis is unknown, but genetic factors are thought to play an important role in the development of the disease.^[21]^The HLA-B27 gene is strongly associated with the risk of developing ankylosing spondylitis, but not all people who carry the gene will develop ankylosing spondylitis.^[22]^ Other environmental and lifestyle factors, such as smoking and infections, may also play a role in the development of ankylosing spondylitis.^[23,24]^ Rheumatoid arthritis is associated with many diseases such as hyperthyroidism, obesity, and cancer.^[25–27]^ Similarly, the present study confirms that RA is positively associated with the risk of ankylosing spondylitis from a genetic point of view. In addition statistical evidence from sensitivity analyses strongly supports our findings. Currently, the mechanism by which RA leads to the development of ankylosing spondylitis is not fully understood. Some studies suggest that RA and ankylosing spondylitis may share some genetic factors, such as HLA-B27.^[28]^ In addition, abnormal activation of inflammatory factors and immune cells may also play an important role in the development of RA leading to ankylosing spondylitis.^[29]^ Therefore, further studies could help to reveal the association between RA and ankylosing spondylitis and provide new strategies for early diagnosis and intervention.

Despite the association between RA and ankylosing spondylitis, the mechanisms and predictors of RA leading to ankylosing spondylitis are not fully understood. In addition, due to the long duration of ankylosing spondylitis, clinical symptoms may not be apparent in the early stages, leading to delays in the diagnosis and treatment of ankylosing spondylitis. Therefore, early identification and intervention in the development of ankylosing spondylitis in patients with RA remains challenging.

There are also some limitations of this study. Firstly, as all data are from people of European origin, the results do not represent a truly randomized population sample and are not applicable to other so races. Secondly, although various sensitivity analyses have been performed in this study to test the hypotheses of the MR study, it is difficult to completely rule out horizontal pleiotropy of instrumental variables. Finally, the current sample size of GWAS data is still not large enough, and more in-depth studies using more GWAS data are needed in the future.

## 5. Conclusion

In conclusion, this study used two-sample MR analysis to analyze and explore the genetic data, and the results showed a causal relationship between RA and ankylosing spondylitis.

## Author contributions

**Conceptualization:** Guang-hua Deng.

**Data curation:** Guang-hua Deng.

**Formal analysis:** Guang-hua Deng.

**Funding acquisition:** Guang-hua Deng.

**Investigation:** Guang-hua Deng.

**Methodology:** Guang-hua Deng.

**Project administration:** Guang-hua Deng.

**Resources:** Guang-hua Deng.

**Software:** Guang-hua Deng.

**Supervision:** Guang-hua Deng.

**Validation:** Guang-hua Deng.

**Visualization:** Guang-hua Deng.

**Writing – original draft:** Guang-hua Deng.

**Writing – review & editing:** Guang-hua Deng.
